# Multidrug-Resistant Pathogens in Hospitalized Syrian Children 

**DOI:** 10.3201/eid2301.161204

**Published:** 2017-01

**Authors:** Diana Faour Kassem, Yoav Hoffmann, Naama Shahar, Smadar Ocampo, Liora Salomon, Zeev Zonis, Daniel Glikman

**Affiliations:** Galilee Medical Center, Nahariya, Israel (D. Faour Kassem, Y. Hoffmann, N. Shahar, S. Ocampo, L. Salomon, Z. Zonis, D. Glikman);; Bar-Ilan University, Safed, Israel (Y. Hoffmann, D. Glikman)

**Keywords:** antibiotic resistance, antimicrobial resistance, bacteria, carriage, infections, pediatric, Syria, Israel, children, multidrug resistance

## Abstract

Since 2013, wounded and ill children from Syria have received treatment in Israel. Screening cultures indicated that multidrug-resistant (MDR) pathogens colonized 89 (83%) of 107 children. For 58% of MDR infections, the pathogen was similar to that identified during screening. MDR screening of these children is valuable for purposes of isolation and treatment.

As the civil war in Syria enters its sixth year, the United Nations estimates that ≈250,000 persons have been killed, ≈10,000 of them children (*1*). Preliminary reports indicate a high rate of multidrug-resistant (MDR) pathogen carriage among refugees from Syria, mostly adults (*2*–*5*). Preliminary data for 29 wounded Syrian children indicate that 66% carried extended-spectrum β-lactamase–producing *Enterobacteriaceae* (ESBL) (*2*).

For ≈3 years, Syrian children who were ill or severely wounded from the civil war have been secretly transported across the border for treatment in Israel, mainly at Galilee Medical Center (GMC; Nahariya, Israel). We characterized carriage of and infections with MDR pathogens among these children.

We prospectively collected demographic and clinical microbiology data for all Syrian children 0–17 years of age who were admitted to GMC during March 2013–February 2016. At admission, contact isolation and screening cultures for MDR were conducted. MDR pathogens belonged to 1 of 5 groups: ESBL, carbapenem-resistant *Enterobacteriaceae* (CRE), methicillin-resistant *Staphylococcus aureus*, MDR *Acinetobacter baumannii* (MDR-AB), and vancomycin-resistant *Enterococcus*. Culture sites included nares, axilla, groin, rectum, and open wounds. Bacterial identification and susceptibility testing were performed according to Clinical and Laboratory Standards Institute guidelines (http://clsi.org/standards/micro/). For CRE screening, we used CHROMagar plates (hylabs, Rehovot, Israel). The mechanism of CRE resistance was determined by the GeneXpert Carba-R system (Cepheid, Sunnyvale, CA, USA) with PCR. For MDR-AB screening, we used MacConkey plates with ceftriaxone disks. The study was approved by the GMC institutional review board.

During the study period, 128 Syrian children were admitted to GMC; 92 (72%) were male, median age was 11 (0–17) years, and 87 (68%) were treated for multiple trauma and 41 (32%) for acute or chronic illness. Average hospitalization length was 1 month. Because of trauma severity, 4 (3%) died at arrival.

Of the 128 children, screening cultures for all 5 pathogen groups were performed for 107, and these children were included in the study (Figure). MDR pathogen carriage was found for 89 (83%). Among MDR pathogens carried, all 5 groups were represented. Among CRE, the most common resistance mechanism was New Delhi metallo-β-lactamase. Rates of MDR carriage were similar among wounded and ill children, mean MDR carriage rate among children increased from 1.19 in 2013–2014 to 1.45 in 2015 (p = 0.02). No amikacin resistance to ESBL was detected. Colistin was the only drug to which CRE and MDR-AB were susceptible.

**Figure:**
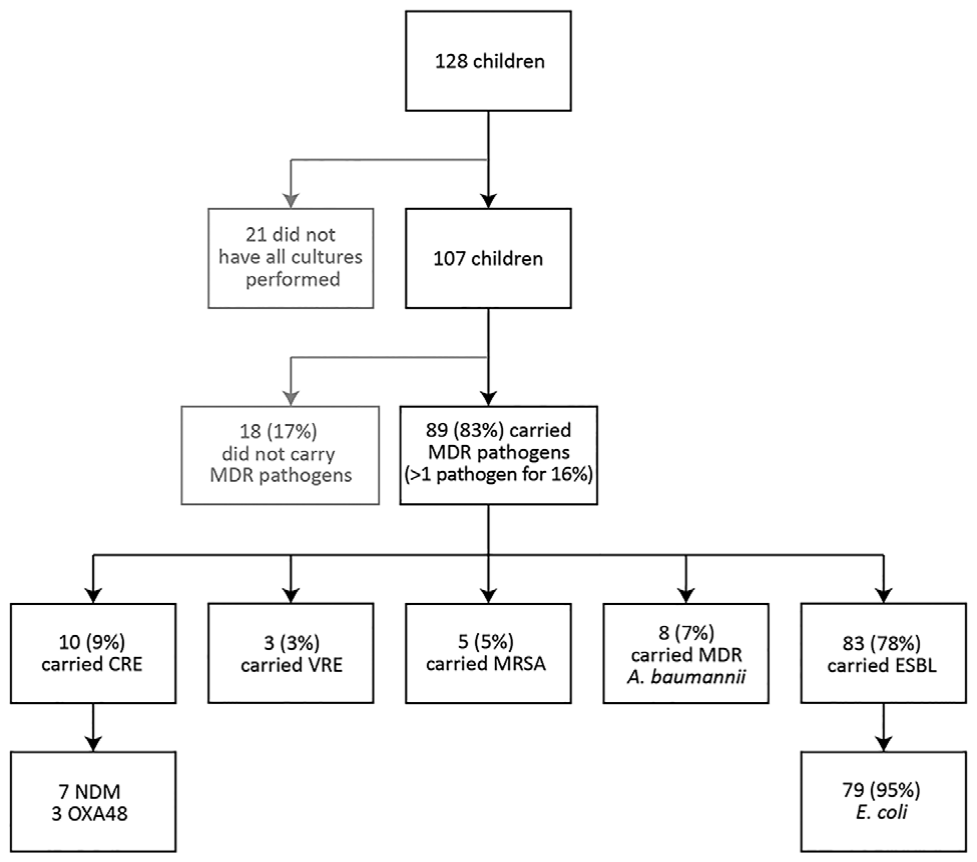
Carriage of MDR pathogens from 5 pathogen groups in 107 Syrian children treated at Galilee Medical Center, Nahariya, Israel, March 2013–February 2016. Of 128 children included in the study, 29 had been previously reported (*2*); all MDR carriage rate calculations are based on the 107 children for whom screening cultures for all 5 pathogen groups were performed. *A. baumannii, Acinetobacter baumannii*; CRE, carbapenem-resistant *Enterobacteriaceae*; *E. coli*, *Escherichia coli*; ESBL, extended-spectrum β-lactamase–producing *Enterobacteriaceae*; MDR, multidrug resistant; MRSA, methicillin-resistant *Staphylococcus aureus*; NDM, New Delhi metallo-β-lactamase; OXA, oxacillinase; VRE, vancomycin-resistant *Enterococcus*.

For 24 (19%) children, MDR infections were evident, mostly urinary tract, surgical site, and osteomyelitis. Infections were more frequent among wounded (90%) than among sick (10%) children (p = 0.035). Two thirds of infections were caused by ESBL, 20% by MDR-AB, and 15% by CRE. In 58% of children with infections, the infecting isolate was similar (by species and antimicrobial drug susceptibility) to the MDR pathogen identified by screening. The empirically prescribed therapy for sepsis was meropenem and amikacin. Colistin was empirically added for severe sepsis or colonization with CRE or MDR-AB.

The rate of MDR carriage among wounded and ill Syrian children who were treated in Israel is extremely high. This finding probably implies a high carriage rate of MDR pathogens (especially ESBL) among healthy children in Syria and acquisition of MDR pathogens (especially CRE and MDR-AB) in the healthcare system in Syria. Indeed, before the Syrian war, prevalence of ESBL urinary tract infections associated with wide use of antimicrobial drugs was high (*6*). In addition, sale of antimicrobial drugs without prescriptions was common in Damascus and other areas (*7*). The war added inadequate sanitation, hospital and infrastructure destruction, and suboptimal infection control measures. Increased rates of MDR pathogen carriage in 2015, compared with 2013–2014, along with the recent polio and measles outbreaks, suggest further deterioration of the healthcare system in Syria (*8*).

Neighboring countries and European centers (for healthcare and asylum seekers) report finding MDR pathogens among wounded adult patients and refugees from Syria; in Germany, among refugees from Syria in 2016, the rate of colonization with gram-negative MDR pathogens was 60% (*3*–*5*,*9*). Earlier in the Syrian conflict, we described an MDR pathogen carriage rate of 66% (almost all ESBL) among 29 children in Israel (*2*). Since then, many more Syrian children have been treated at GMC. Approximately 80% of these children are colonized by MDR pathogens; ESBL predominate, followed by CRE and MDR-AB*.*

Our data, relevant to other centers caring for patients from Syria, show that contact isolation at the time of admission of patients from Syria is crucial for preventing transmission of MDR pathogens. We did not observe cross-infections, but another center in Israel that cares for patients from Syria has documented nosocomial spread of oxacillinase-48 CRE (*10*). Thoughtful decisions about empiric antimicrobial drug therapy for patients from Syria suspected of having infection are complex, given the multitude of MDR pathogens carried and the severity of trauma. Our study demonstrates that in almost 60% of MDR infections, the pathogen was similar to that found on screening cultures. Therefore, screening cultures at admission can provide valuable information for infection control, isolation decision-making, and tailoring appropriate empiric antimicrobial drug therapy.

## References

[1] United Nations. Alarmed by continuing Syria crisis, Security Council affirms its support for Special Envoy’s approach in moving political solution forward [cited 2016 Jun 9]. http://www.un.org/press/en/2015/sc12008.doc.htm

[2] Peretz A, Labay K, Zonis Z, Glikman D. Disengagement does not apply to bacteria: a high carriage rate of antibiotic-resistant pathogens among Syrian civilians treated in israeli hospitals. Clin Infect Dis. 2014;59:753–4. 10.1093/cid/ciu374 24846634

[3] Rafei R, Dabboussi F, Hamze M, Eveillard M, Lemarié C, Mallat H, et al. First report of *bla*_NDM-1_-producing *Acinetobacter baumannii* isolated in Lebanon from civilians wounded during the Syrian war. Int J Infect Dis. 2014;21:21–3. 10.1016/j.ijid.2014.01.004 24560830

[4] Reinheimer C, Kempf VAJ, Göttig S, Hogardt M, Wichelhaus TA, O’Rourke F, et al. Multidrug-resistant organisms detected in refugee patients admitted to a University Hospital, Germany June‒December 2015. Euro Surveill. 2016;21:30110. 10.2807/1560-7917.ES.2016.21.2.30110 26794850

[5] Angeletti S, Ceccarelli G, Vita S, Dicuonzo G, Lopalco M, Dedej E, et al.; Sanitary Bureau of Asylum Seekers Center of Castelnuovo di Porto. Unusual microorganisms and antimicrobial resistances in a group of Syrian migrants: Sentinel surveillance data from an asylum seekers centre in Italy. Travel Med Infect Dis. 2016;14:115–22. 10.1016/j.tmaid.2016.03.005 26987764

[6] Al-Assil B, Mahfoud M, Hamzeh AR. First report on class 1 integrons and trimethoprim-resistance genes from *dfrA* group in uropathogenic *E. coli* (UPEC) from the Aleppo area in Syria. Mob Genet Elements. 2013;3:3,e25204. 10.4161/mge.25204 PMC3742597 23956949

[7] Al-Faham Z, Habboub G, Takriti F. The sale of antibiotics without prescription in pharmacies in Damascus, Syria. J Infect Dev Ctries. 2011;5:396–9. 10.3855/jidc.1248 21628818

[8] Sharara SL, Kanj SS. War and infectious diseases: challenges of the Syrian civil war. PLoS Pathog. 2014;10:e1004438. 10.1371/journal.ppat.1004438 25393545 PMC4231133

[9] Teicher CL, Ronat JB, Fakhri RM, Basel M, Labar AS, Herard P, et al. Antimicrobial drug-resistant bacteria isolated from Syrian war-injured patients, August 2011-March 2013. Emerg Infect Dis. 2014;20:1949–51. 10.3201/eid2011.140835 25340505 PMC4214314

[10] Lerner A, Solter E, Rachi E, Adler A, Rechnitzer H, Miron D, et al. Detection and characterization of carbapenemase-producing *Enterobacteriaceae* in wounded Syrian patients admitted to hospitals in northern Israel. Eur J Clin Microbiol Infect Dis. 2016;35:149–54. 10.1007/s10096-015-2520-9 26581423

